# Development of a Markerless Deletion System for the Fish-Pathogenic Bacterium *Flavobacterium psychrophilum*

**DOI:** 10.1371/journal.pone.0117969

**Published:** 2015-02-18

**Authors:** Esther Gómez, Beatriz Álvarez, Eric Duchaud, José A. Guijarro

**Affiliations:** 1 Área de Microbiología, Departamento de Biología Funcional, Facultad de Medicina, IUBA, Universidad de Oviedo, 33006, Oviedo, Spain; 2 Facultad de Ciencias de la Salud, Universidad Autónoma de Chile, Santiago de Chile, Chile; 3 Virologie et Immunologie Moléculaires UR892, INRA (Institut National de la Recherche Agronomique), 78350, Jouy-en-Josas, France; University of Minnesota, UNITED STATES

## Abstract

*Flavobacterium psychrophilum* is a Gram-negative fish pathogen that causes important economic losses in aquaculture worldwide. Although the genome of this bacterium has been determined, the function and relative importance of genes in relation to virulence remain to be established. To investigate their respective contribution to the bacterial pathogenesis, effective tools for gene inactivation are required. In the present study, a markerless gene deletion system has been successfully developed for the first time in this bacterium. Using this method, the *F. psychrophilum* *fcpB* gene, encoding a predicted cysteine protease homologous to *Streptococcus pyogenes* streptopain, was deleted. The developed system involved the construction of a conjugative plasmid that harbors the flanking sequences of the *fcpB* gene and an I-*Sce*I meganuclease restriction site. Once this plasmid was integrated in the genome by homologous recombination, the merodiploid was resolved by the introduction of a plasmid expressing I-*Sce*I under the control of the *fpp2 F. psychrophilum* inducible promoter. The resulting deleted *fcpB* mutant presented a decrease in extracellular proteolytic activity compared to the parental strain. However, there were not significant differences between their LD_50_ in an intramuscularly challenged rainbow trout infection model. The mutagenesis approach developed in this work represents an improvement over the gene inactivation tools existing hitherto for this “fastidious” bacterium. Unlike transposon mutagenesis and gene disruption, gene markerless deletion has less potential for polar effects and allows the mutation of virtually any non-essential gene or gene clusters.

## Introduction

*Flavobacterium psychrophilum* is the etiological agent of the bacterial cold-water disease (BCWD) and rainbow trout fry syndrome (RTFS), which particularly affects juvenile rainbow trout *(Oncorhynchus mykiss)*, causing important economic losses in salmonid aquaculture worldwide. The disease mainly appears when water temperatures range between 10°C and 14°C [1] and, as there is no commercial vaccine, its control requires the massive use of antibiotics.

It is considered a “fastidious” bacterium because it is difficult to isolate and manipulate [2]. Therefore, the knowledge regarding its virulence factors is still fairly limited. In this respect, some factors associated with pathogenesis have been described. Among these, an iron acquisition system [3], adhesion ability [4], hemolytic activity [5–7] and a thiol:disulfide oxidoreductase [8] were reported to play role in virulence. Predicted virulence genes have also been identified by genomic analysis [9] and extracellular proteases have been proposed to play major function in the virulence of the bacterium [10, 11]. However, two of them (Fpp1 and Fpp2) were suggested to have a nutritional role [12].

The study of virulence factors of *F. psychrophilum* has been drastically hampered by the difficulty to genetically manipulate this organism. Transformation and conjugation frequencies are very low and strain specific [13], growth is slow [14] and recovery of cells on solid media comes up against the presence of viable but non-culturable cells [15]. In spite of this, it is worth to highlight the development, in the last few years, of a transposon mutagenesis technique [13] and a GFP-based promoter probe vector [16]. Furthermore, mutagenesis via single-crossover homologous recombination was successfully used for the mutation of the *fpp1* and *fpp2* genes [12] as well as other genes (Gomez et al., unpublished results). Nevertheless, this technique presents some potential limitations such as the generation of polar mutations, obtaining partially-inactivated proteins and reversibility of mutations. Therefore, additional tools are still needed in order to improve the genetic manipulation in this microorganism. The principal objective of this work was to develop a deletion mutagenesis method for *F. psychrophilum*, based on the one designed by Pósfai et al. (1999) [17] for *Escherichia coli*, which has been also used in *Bacteroides fragilis* [18], a species phylogenetically more closely related to *F. psychrophilum* and other bacteria [19–21]. The system is based on the I-*Sce*I meganuclease, a restriction enzyme recognizing an 18 bp sequence that serves as a stimulator of the recombination process [17]. Briefly, a suicide plasmid, which carries the flanking sequences of the gene to be deleted and the recognition site of I-*Sce*I, is inserted into the genome by homologous recombination between the flanking sequences of the gene to be deleted and those matching sequences in the chromosome of the bacterium. Resolution of this cointegrate via intramolecular recombination, which is stimulated by the action of the I-*Sce*I meganuclease, generates either mutant or wild type chromosome. The I-*Sce*I coding sequence is present in a replicative plasmid that is introduced into the merodiploid clones and its expression is regulated by the calcium-temperature inducible promoter of the gen *fpp2* [16].

The *fcpB* gene of *F. psychrophilum* THCO2/90 was selected for being deleted. This locus is specifically present in the genome of this virulent strain and codes for a protein homologous to the cysteine protease “streptopain”, a major virulence determinant in *Streptococcus pyogenes* [22–26]. ScpB is a critical virulence factor for invasive disease episodes [22, 23]. It also destroys most of signaling and antibacterial properties of chemokines expressed by an inflamed epithelium and increases capillary permeability and histamine release from mast cells [24]. All of these properties, amongst others, make ScpB a major virulence determinant of *S. pyogenes*. Therefore, we hypothesize that the FcpB protein, which is homologous to ScpB, could have some relevance in the pathogenic process of *F. psychrophilum* THC02/90. The additional objective was then to know the implication of this gene in the pathogenic process of *F. psychrophilum* in a rainbow trout infection model.

## Materials and Methods

### Bacterial strains, plasmids, growth conditions and proteolytic activity

*Escherichia coli* strain S17–1λpir [27] was grown at 37°C in 2xTY medium (10 g tryptone, 10 g yeast extract, 5 g NaCl per liter) with 20 g/l agar added for solid medium. This strain was used to transfer DNA into *F. psychrophilum*. The *F. psychrophilum* THC02/90 strain was grown at 12°C or 18°C in nutrient broth (NB; Pronadisa; 5 g gelatin peptone, 3 g beef extract per liter) or nutrient broth containing 10 mM CaCl_2_ (NBF) [28]. Nutrient agar (NA; NB containing 15 g/l agar) or nutrient agar charcoal [NAC; NA supplemented with activated charcoal (Sigma)] were used for solid cultures, as previously described [29]. For selective growth of *E. coli* S17–1 λpir, 50 μg/ml streptomycin was used and transformants were selected with 100 μg/ml ampicillin. Selection of *F. psychrophilum* transconjugants was carried out with 10 μg/ml erythromycin or 10 μg/ml tetracycline. The plasmids and primers used are listed in Table 1. Colony spreading was analyzed according to Pérez-Pascual et al. (2010) [30]. Briefly, plates containing one-sixth NA were inoculated in their center with 8 μl liquid culture in middle exponential growth phase. The plates were then incubated at 20°C, and spreading and biomass production was quantified at 120 h by measuring the colony diameter (mm) and OD_525_ of 1 ml aqueous suspensions of colonies, respectively. These experiments were performed in triplicate. Azocasein assays using supernatants of liquid cultures were carried out according to Secades et al. (2001) [28]. One unit of enzyme activity (enzymatic units; EU) was defined as the amount of enzyme which yielded an increase in the A_420_ of 0.01 in 2 h at 30°C. The protease activity assays were performed in triplicate (supernatants were taken from 3 different cultures of each strain) and the protease activity was measured in triplicate for each sample.

**Table 1:** Strains, plasmids and oligonucleotides used in this work.

Strain, plasmid or primer	Description or sequence*	Reference
**Strains**		
*E. coli*		
S17–1 λ *pir*	λ*pir hsdR pro thi*; RP4–2 Tc::Mu Km::Tn7	[27]
*F. psychrophilum*		
THC02/90	Parental strain	[36]
THC02/90–23	Parental strain carrying pCP23 plasmid	This study
THC02/90-TFS	Parental strain carrying pCP23-TFS plasmid	This study
fcpB3+	Merodiploid strain resulting from overcrossing between del3’ fragments	This study
fcpB5+	Merodiploid strain resulting from overcrossing between del5’ fragments	This study
fcpB^-^	FpcB^-^ mutant	This study
**Plasmids**		
pCP23	ColE1 ori; (pCP1 ori), Ap^r^ (Tc^r^), bifunctional plasmid *E*.*coli-F*. *psychrophilum*	[37]
pCP23-Gfpp2	pCP23-G plasmid carrying Pfpp2 inducible promoter; Ap^r^ (Tc^r^)	[16]
pLYL03	ColE1 ori; RK2oriT; Ap^r^ (Em^r^).	[32]
Pacbsr	Plasmid carrying I*Sce*-I coding sequence	[38]
pCP23-TFS	pCP23 plasmid carrying a trancription terminator (T), an inducible promoter (Pfpp2) and I*Sce*-I coding sequence; Ap^r^ (Tc^r^)	This study
pLYL03–3S5	pLYL03 plasmid carrying 1.5 kb *fcpB* flanking sequences and I*Sce*-I restriction site; Ap^r^ (Em^r^)	This study
**Oligonucleotides**		
promfpp2_F	5’ ATCA**GGATCC**GAGCACTACACTTTCTAGA 3’	This study
pfpp2_Sce_R	5’TTGATGTTTTTCATATGCATATGTATATCTCCTTCTTAAA**AGATCT**TGTTCGGTAGTGTAGC 3’	This study
fus_Sce_F	5’AGATCTTTTAAGAAGGAGATATACATATGCATATGAAAAACATCAAAAAAAACCAGGTAATG 3’	This study
sce_R	5’ CTCCTTA**GCATGC**CTGCAGACGTCG 3’	This study
RP	5’ GAGGAAACAGCTATGAC 3’	This study
fcpBdel3’_F	5’ ATCG**TCTAGA**TGGTTGGTATAATGCTC 3’	This study
fcpBdel3’_R	5’ ATCG**GGATCC**TTTGGTGAAGATGAAATTA 3’	This study
fcpBdel5’_F	5’ATCG**CTGCAG** TAGGGATAACAGGGTAACGTATTTTAGGATAAGAC 3’	This study
fcpBdel5’_R	5’ ATCG**TCTAGA**TTGCGAGAATAAAATTTTTA 3’	This study
fcpBint_F	5’ AAATCAACATGAAGTAACACAA 3’	This study
fcpBint_R	5’ CCAGTTCATGTGTAAATATAGAT 3’	This study
RTSceI_F	5’ AAACTGCTGAAAGAATACAAATC 3’	This study
RTSceI_R	5’ AGGAGATAGTGTTCGGCAGT 3′	This study

* Antibiotic-resistance phenotypes: Ap^r^, ampicillin; Tc^r^, tetracycline; Em^r^, erythromycin. Antibiotic-resistance phenotypes and other features listed in parentheses are those expressed by *F. psychrophilum* but not by *E. coli*.

Restriction sites are in bold type. I*Sce*-I restriction site is underlined.

### DNA procedures

Plasmid DNA was isolated using the “Gen Elute Plasmid miniprep” commercial kit and genomic DNA was purified with the “Gen Elute Bacterial Genomic DNA” kit, both from Sigma Aldrich Co., Switzerland. Routine DNA procedures such as DNA digestion with restriction enzymes, DNA ligations, and gel electrophoresis were performed essentially as described by Sambrook et al. (2001) [31]. Restriction enzymes were from Takara Bio Co., Japan. All PCR products were amplified using Pfu polymerase (New England Biolabs, MA) according to the manufacturer recommendations. The oligonucleotides used in this study are described in Table 1. T4 DNA ligase was from Roche Diagnostics GmbH, Germany.

### Generation of the *fcpB* ^-^ deletion mutant

Flanking sequences of the *fcpB* gene of approximately 1.5 kb each were amplified by PCR. Flanking regions were cloned consecutively into the multiple cloning site of pLYL03 plasmid (Table 1). The 3′ *fcpB* flanking sequence was amplified with the oligonucleotides FcpBdel3’_F and FcpBdel3’_R (Table 1) and cloned into the *BamH*I-*Xba*I sites whereas the 5′ *fcpB* flanking sequence was amplified with the primers FcpBdel5’_F and FcpBdel5’_R and cloned into the *XbaI-PstI* sites. The FcpBdel5’_ F primer contained an I-*Sce*I restriction site. The resulting plasmid, named pLYL03–3S5, was transferred to *F. psychrophilum* THC02/90 from *E. coli* S17–1λpir by conjugation as described by Álvarez et al. (2004) [13] and transconjugants were selected in NAC with 10 μg/ml erythromycin. One hundred microliters of NB with erythromycin were inoculated with each clone in microtiter plates and those that showed superior growth were selected to verify, by PCR and Southern Blot, if they had the plasmid integrated into the chromosome. PCRs were performed with RP and FcpBdel5’ primers (Table 1), using genomic DNA from the different strains as template. Genomic DNA from *F. psychrophilum* THC02/90 and pLYL03–3S5 plasmid were used as negative and positive controls, respectively. To perform Southern Blot, *Hind*III digested genomic DNA was hybridized with a 970 bp DIG-labelled PCR probe (DIG DNA labelling mix, Roche Diagnostics GmbH, Germany) using the oligonucleotides FcpBdel5’_F and FcpBdel5’_R and genomic DNA from the parental strain as template. Strains with the plasmid pLYL03–3S5 integrated into the chromosome were named FcpB5^+^, if the recombination has occurred in the 5’ flanking sequence, and FcpB3^+^, if it has occurred in the 3’ flanking sequence (Table 1).

A replicative plasmid, derived from pCP23 (Table 1), was constructed to facilitate the next over-crossing event. The calcium and temperature-inducible promoter Pfpp2 from the *fpp2* gene [16] was fused to the I-*Sce*I coding region by cross-over PCR. The promoter sequence was amplified with the primers promfpp2_F and pfpp2_Sce_R (Table 1) and I-*Sce*I coding sequence was amplified with Fus_Sce_F and Sce_R (Table 1), using the plasmids pCP23-Gfpp2 and pACBSR (Table 1) as templates, respectively. Both fragments were used as templates in a cross-over PCR with the primers promfpp2_F and Sce_R (Table 1), to obtain the fused fragment of 1.3 kb containing the Pfpp2 promoter in front of the I-*Sce*I coding region (Pfpp2-SceI), This fragment was cloned into the plasmid pCP23-Gfpp2, previously digested with *Bam*HI and *Sph*I enzymes to replace the Pfpp2-gfpmut3 region for the fused Pfpp2-SceI fragment. The resulting plasmid was named pCP23-TFS. The pCP23-TFS plasmid was transferred from *E. coli* S17 1 λpir to *F. psychrophilum* FcpB5^+^ or FcpB3^+^ by conjugation as described by Álvarez et al. (2004) [13]. Transconjugants were selected in NAC with tetracycline and 10 mM CaCl_2_ and incubated at 12°C. One hundred microliters of NB with tetracycline were inoculated with each clone in microtiter plates and those that showed superior growth (because they carried the pCP23-TFS plasmid) were selected to confirm, by PCR and Southern Blot, if the deletion of the *fcpB* gene had ocurred. PCR was performed, using the primers FcpBint_F and FcpBint_R (Table 1), and genomic DNA from *F. psychrophilum* THC02/90, FcpB5^+^ and FcpB3^+^ as controls. To confirm the *fcpB* gene deletion by Southern Blot, genomic DNA from *F. psychrophilum* THC02/90 and *fcpB* ^-^ mutant was digested with *Hind*III and hybridized with a 970 bp DIG-labelled PCR probe using the oligonucleotides FcpBint_F and FcpBint_R and genomic DNA from the parental strain as template.

### Effect of pCP23-TFS in *F. psychrophilum* growth and I-*Sce*I RT-PCR

The absence of I-*Sce*I restriction sequences in the *F. psychrophilum* THC02/90 genome was assessed by *in silico* analysis. However, in order to discard any potential negative effect of I-*Sce*I expression in *F. psychrophilum*, the plasmid pCP23-TFS was transferred from *E. coli* S17 1–λpir to *F. psychrophilum* THC02/90 by conjugation. Two hundred and fifty microliters of the resulting strain, named THC02/90-TFS (Table 1), was then inoculated to 25 ml of NB in 250 ml flasks and incubated at 12°C with shaking. The parental strain was used as a control. The optical density at 525 nm of both cultures was measured with a Hitachi U2900 Spectrophotometer over time. RT-PCR. Total RNA was obtained from 2 ml of early stationary phase cultures of *F. psychrophilum* THC02/90–23 (Table 1) and THC02/90-TFS strain, which were grown in NBF supplemented with 5 μg/ml tetracycline, at 12°C. RNA was isolated using a High Pure RNA Isolation kit (Roche) and treated with DNase I (RNase-free) (Ambion) to eliminate traces of DNA. Reverse transcription (RT-PCR) was performed using Superscript One-Step with Platinum Taq System (Invitrogen Life Technologies), using 20 ng of RNA in each reaction. In order to determine whether RNA was free of contaminant DNA, reactions omitting the reverse-transcription step were included in each run as negative controls. The primers used for RT-PCR, which target an intragenic region of I-*Sce*I coding sequence, were RTSceI_F (nucleotides 54–77) and RTSceI_R (nucleotides 674–694). RT-PCR program was set following manufacturer’s indications, with an annealing temperature of 62°C and an elongation time of 50 s.

### LD_50_ determinations

Animal experiments were performed in accordance with the European legislation governing animal welfare, and they were authorized and supervised by the Animal Experimentation Ethics Committee of Universidad de Oviedo (see S1 Information). Rainbow trout (*O. mykiss*) fingerlings weighing between 5 and 7 g used for the animal experiments were obtained from a commercial fish farm. Fish were acclimatized to experimental conditions and randomly selected fish were analysed to discard the presence of bacteria in spleen, gut and liver. Fish were kept in 60 l tanks at 12±1°C in continually flowing dechlorinated water. For LD_50_ determinations, *F. psychrophilum* cultures were grown to exponential phase, harvested by centrifugation and washed with PBS. Cells were resuspended in PBS and serial dilutions were prepared. Groups of 10 fish were challenged by intramuscular injection with 50 μl of dilutions containing 10^3^–10^5^ CFU and they were monitored twice daily. LD_50_ was calculated according to the PROBIT method using IBM SPSS Statistics 19.0 (Armonk, New York, USA), establishing a 95% confidence limit. LD_50_ experiments were performed in duplicate.

### In silico analysis

FcpB (*Flavobacterium* Cysteine protease B) gene sequence was obtained from the ongoing genome project of *F. psychrophilum* THC02/90 (Duchaud E., unpublished data). The National Center for Biotechnology Information (NCBI) Basic Local Alignment Search Tool (BLAST) was used to compare protein sequences and Simple Modular Architecture Research Tools (SMART) to detect conserved domains. MotifScan software from MyHits was used to identify the motifs present in each sequence. The ProtParam program (ExPASy) was used for molecular mass computation and SignalP3.0 (Center for Biological Sequence Analysis; CBS) to predict the location of a signal peptide cleavage site. The MEROPS peptidase database was used to classify the protease. Accession number of *fcpB* gene is GenBank KJ605411.

## Results

### Analysis of the *fcpB* gene of *F. psychrophilum* THC02/90

**Fig 1:**
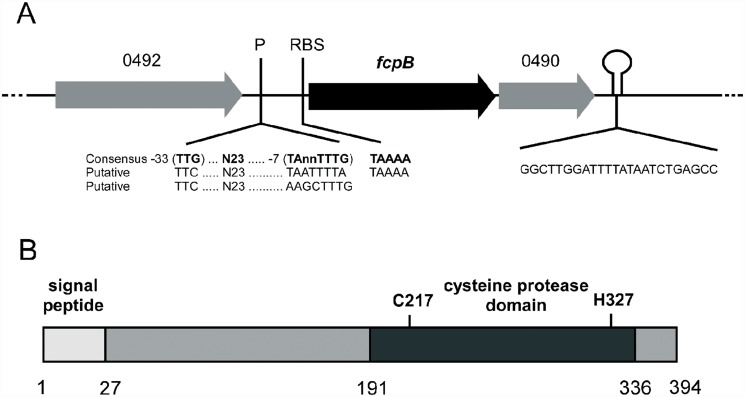
*fcpB* locus and FcpB predicted structure. (A) Gene organization of the fcpB locus in the *F. psychrophilum genome*. The position of each gene and the direction of the transcription are shown by arrows. The putative *fcpB* promoters (P), ribosomal binding site (RBS), and rho-independent transcriptional terminator (hairpin loop), together with the corresponding sequences, are showed. The flanking genes 0492 (encoding a putative transmembrane protein) and 0490 (encoding a putative lipoprotein precursor) are indicated. (B) Structural organization and predicted domains of the FcpB cystein peptidase. Domain regions are dark grey and the position of the initial and final residues of each domain are indicated below. The amino acids corresponding to the active site are indicated in bold.

The *fcpB* gene (locus THC0290–0491) is encompassed in a large genomic island, specific of the THC02/90 strain (Duchaud, unpublished results). This region has an unusually high proportion of genes of unknown function and also contains an important number of transposases-encoding genes belonging to different families (Duchaud, unpublished results). The *fcpB* gene is flanked by the loci 0492 and 0490 encoding a putative transmembrane protein of unknown function and a hypothetical lipoprotein precursor, respectively. Putative promoter and terminator sequences were also identified (Fig. 1A).

The *fcpB* gene is predicted to encode a 394 amino-acid protein with significant homology with bacterial cysteine endopeptidases such as streptopains and others C10 family peptidases from different bacterial species including *Dyadobacter fermentans* (identity 32%; E-value 4e-45), *Spirosoma linguale* (identity 37%; E-value 1e-44), *Bacteroides intestinalis* (identity 34%, E-value 2e-32), *S*. *pyogenes* (identity 28%, E-value 4e-13) and *Flavobacterium branchiophilum* (identity 26%, E-value 2e-15). FcpB seems to be an extracellular protein since it contains a predicted signal peptide of 27 amino acids (Fig. 1B). It also has a peptidase C10 family domain located from amino acid 191 to 336 (Fig. 1B). In addition, FcpB as other bacterial cysteine endopeptidases, seems to be synthesized as a proenzyme, having a predicted pro-peptide that blocks the active site of the enzyme corresponding to the C217 amino acids position to H327 (Fig. 1B).

### Generation of a gene deletion in *F. psychrophilum*

**Fig 2:**
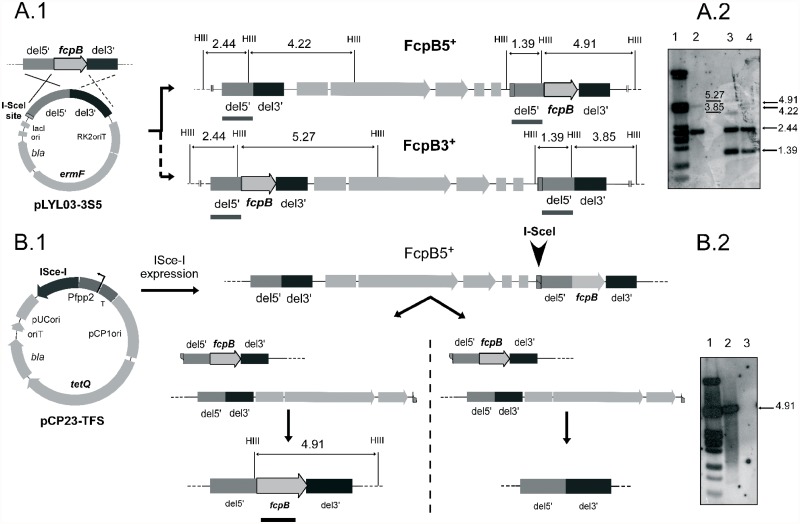
Markerless deletion strategy for *F. psychrophilum*. (A1) A suicide plasmid (pLY03–3S5), containing an I-*Sce*I recognition site and two *fcpB* flanking sequences of 1.5 kb (del5’ and del3’), was introduced in *F. psychrophilum* THC02/90 by conjugation. RK2oriT: conjugal transfer origin; *ermF*: erythromycin resistance gene in *F. psychrophilum*; *bla*: ampicillin resistance gene in *E. coli*; ori: replication origin in *E. coli*; lacI: *lacI* gene. Two possible integrations, depending on the flanking region where the recombination had occurred (FcpB5+ and FcpB3+ strains), are represented following continuous and dashed lines. *Hind*III restriction sites are indicated with HIII and the size of Southern Blot expected bands, using del5´ as a probe (grey line), are indicated above each scheme. (A2) Southern blot hybridization using a PCR generated labeled del5´ DNA fragment as a probe. Genomic DNA, from the parental strain *F. psychrophilum* THCO2/90 (lane 2) and the transconjugants which harbor the two possible generated cointegrates: lanes 3, FcpB3^+^ and lane 4, FcpB5^+^, was digested with *Hind*III and hybridized with del5´ fragment labeled probe. Molecular mass ladder, phage λ DNA digested with *Pst*I (lane 1). The two top hybridization bands corresponding to lane 3 (5.27 and 3.85 kb) and lane 4 (4.91 and 4.22 kb) are tenuous because the length of their homologous sequences with the probe is very short. (B1) The FcpB5^+^ and FcpB3^+^ merodiploid strains were conjugated (only FcpB5^+^ is represented) with an *E. coli* S17–1 λ pir strain harboring a plasmid (pCP23-TFS) expressing I-*Sce*I (I-SceI) under the control of the *F. psychrophilum fpp2* calcium-temperature inducible promoter (Pfpp2). T: transcription terminator; pCP1ori: replication origin in *F. psychrophilum*; *tetQ*: tetracycline resistance gene in *F. psychrophilum*; *bla*: ampicillin resistance gene in *E. coli*; oriT: conjugal transfer origin; pUCori: replication origin in *E. coli*. Transconjugants were selected at 12°C in a medium containing tetracycline and CaCl_2_ leading to the resolution of the merodiploid by generation of a wild-type genotype (left side of the figure) or in a markerless *fcpB* gene deleted strain (right side of the figure). I-*Sce*I mediated double strain break is indicated by an arrow head symbol. (B2) Southern Blot hybridization using genomic DNA from the wild-type and the *fcpB* deleted mutant strain, digested with *Hind*III and hybridized with an internal *fcpB* fragment. Lane 2, wild-type; lane 3 *fcpB* deleted mutant strain; Lane 1, molecular mass ladder (phage λ DNA digested with *Pst*I).

**Fig 3:**
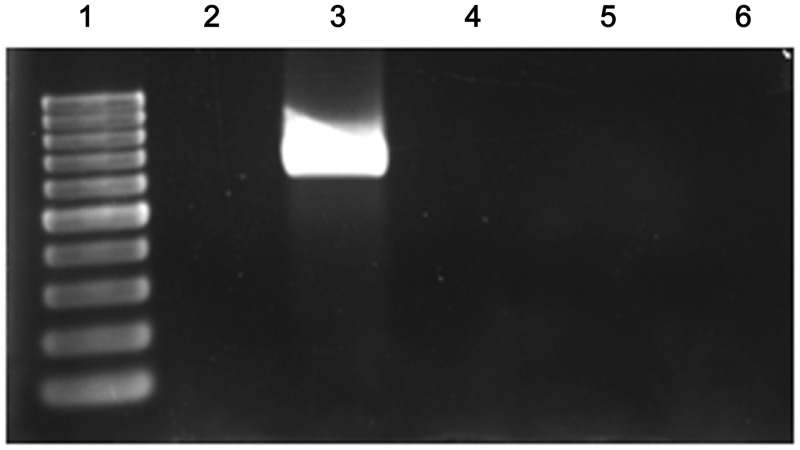
RT-PCR analysis of the I-*Sce*I expression under the CaCl_2_-temperature inducible promoter Pfpp2. Each PCR was performed with 20 ng of *F. psychrophilum* RNA. Oligonucleotides homologous to I-*Sce*I gene, giving an internal RT-PCR fragment of 640 nt, were used. RT-PCRs were performed with RNA from *F. psychrophilum* THC02/90 strain carrying pCP23 plasmid (lane2) and pCP23-TFS plasmid (lane 3), which were incubated at 12°C and CaCl_2_ until early stationary phase. As controls for DNA contamination, PCRs were also carried out with the primers used for RT-PCR analysis by omitting the reverse transcription step, using RNA samples from *F. psychrophilum* THC02/90 strain carrying pCP23 plasmid (lane 4), pCP23-TFS plasmid (lane 5) and DEPC water as templates. Lane 1: 100 bp molecular marker (Biotools).

The genome of *F. psychrophilum* THC02/90 does not have any I-*Sce*I restriction site as revealed by *in silico* analysis. The procedure used to obtain a markerless *fcpB* deletion in *F. psychrophilum* THC02/90 was based on the method of Pósfai et al. (1999) [17]. The conjugative and integrative plasmid pLYL03–3S5 harboring 1.5 kb flanking sequences of *fcpB* and an I-*Sce*I restriction site (Fig. 2A.1) was transferred to *F. psychrophilum* THC02/90 from the strain *E. coli* S17–1λpir by conjugation. Transconjugants were selected on NAC plates with erythromycin and the 3% of the transconjugants (12 out of 400 transconjugants grown on NB with erythromycin in microtiter plates) harbored the plasmid integrated into the chromosome (Fig. 2A.1). This was determined by PCR (data not shown), and Southern Blot, which showed the two expected band patterns corresponding to the two possible integrations depending on the flanking region where the recombination had occurred (Fig. 2A.2). The two merodiploid strains obtained were named FcpB3^+^ and FcpB5^+^ (Fig. 2A.1). To resolve the merodiploids, the conjugative pCP23-TFS plasmid that expresses the I-*Sce*I meganuclease under the control of the *F. psychrophilum fpp2* gene promoter [16] was constructed. This *fpp2* gene promoter is induced at 12°C in the presence of CaCl_2_ [16]. In order to determine whether or not the presence of this plasmid could modify the bacterial physiology it was introduced into the *F. psychrophilum* THC02/90 strain. The growth curve of THC02/90-TFS strain was similar to that of the parental (data not shown). Additionally, transcription of the I-*Sce*I gene under the Pfpp2 promoter in the induction conditions was demonstrated by RT-PCR analysis (Fig. 3).

The pCP23-TFS plasmid was then introduced by conjugation into the merodiploids FcpB3^+^ and FcpB5^+^, and the resulting transconjugants were incubated on a medium containing tetracycline, to maintain the plasmid, and 12°C in the presence of CaCl_2_, to induce the I-*Sce*I expression (Fig. 2B.1). I-*Sce*I activity should mediate a unique double-strand break at its recognition site and homologous recombination repair should allow resolution of the merodiploids and vector loss (Fig. 2B.1). Transconjugants that showed a better growth in NB with tetracycline, in microtiter plates, were screened by PCR for the absence of *fcpB*. One out of 50 transconjugants resistant to tetracycline was PCR negative (2%). Southern Blot using a 970 bp labeled internal fragment of the *fcpB* gene as a probe showed the absence of a 4.9 kb hybridization band, indicating the complete deletion of *fcpB* (Fig. 2B.2).

### Phenotypic characterization and LD_50_ determinations

To characterize the *F. psychrophilum fcpB* ^-^ strain, some of its phenotypic traits were studied. The growth of *fcpB* ^-^ mutant in NBF at 12°C was similar to that of the parental strain (data not shown). However, the determination of the extracellular proteolytic activity during growth indicated that *fcpB* gene contributed to the caseinolytic activity of the bacterium, reaching a maximum difference of about 30% less activity in the mutant fcpB^-^ in comparison with the parental strain after 140 h of incubation (THC02/90: 121.20 ± 0.18 UE ml^-1^; fcpB^-^: 83,94 ± 0,66 UE ml^-1^). Analysis of colony spreading on diluted NA medium showed that fcpB^-^ presented a colony diameter similar to that of the parental strain and both colonies had practically the same biomass production (data not shown).

In order to evaluate the effect of the *fcpB* gene deletion on the virulence of the bacterium, LD_50_ experiments were carried out on rainbow trout. The medium LD_50_ 10 days post-injection was calculated to be 1.48 × 10^5^ CFU, with a lower bound of 9.08 × 10^4^ CFU and an upper bound of 2.51 × 10^5^ CFU, for the parental strain. Under the same conditions, fcpB^-^ showed a LD_50_ value of 1.70 × 10^5^ CFU, with a lower bound of 1.01 × 10^4^ CFU and an upper bound of 2.86 × 10^5^ CFU (Table A in S1 Information). Furthermore, macroscopically examination of skin lesions usually produced around the injection site, revealed that they were similar in both parental and *fcpB* mutant strain (data not shown).

## Discussion

*F. psychrophilum* is a member of the *Cytophaga-Flavobacterium-Bacteroides* (CFB) group and is very distantly related to organisms with well-developed genetic systems, such as members of the proteobacteria. In general, plasmids, selectable markers, and transposons that function in proteobacteria fail to work in members of the CFB group. Techniques to genetically manipulate these bacteria have been developed [32–34]. In particular, Tn*4351*-based transposon mutagenesis [13] and a site-specific mutagenesis method by homologous recombination [12] have been useful in *F. psychrophilum* in order to study virulence determinants. All these studies have decisively contributed to increase the knowledge of the genetic and physiology of this “fastidious” microorganism. Taking into account the limitations of these systems for some studies, a deletion mutagenesis method has been developed in the present work. Two major determinants were important in this procedure: i) large sequences of about 1.5 kb flanking the *fcpB* gene were used to maximize the probability that the first cross-over recombination event took place, ii) the expression of the I-*Sce*I meganuclease gene under the inducible *fpp2* promoter [16] to facilitate the second recombination event to obtain the *fcpB* deletion mutant. It has been described that double-strand breaks mediated by I-*Sce*I enhances the resolution of the merodiploid generating the two possible genotypes: mutant or wild-type [17]. A similar method was used by Patrick et al. (2009) [18], for deletion of two genes involved in LPS and capsular polysaccharide biosynthesis of *B*. *fragilis*. Although the efficiency of obtaining double recombination mutants in *F. psychrophilum* was very low, this mutagenesis strategy was proved to be feasible in this bacterium. Both steps of this markerless deletion system had similar conjugation frequencies than that observed in other previous single-crossover recombination experiments (*fpp1* and *fpp2*, [12]), but having the advantage of reducing or even avoiding the possibility to obtain polar effects. As it occurs with site-directed and transposon mutagenesis systems, conjugation and recombination frequencies are the principal bottleneck to succeed in all these procedures [12, 13]. Moreover, it should be pointed out that, in this bacterium, it is usual to find transconjugants lacking antibiotic resistance determinants. In conclusion, factors that influence conjugation and recombination efficiencies are still not completely known in *F. psychrophilum* and might be improved in the future. So far, this can only be solved by increasing the number of conjugation experiments.

The *fcpB* gene from *F. psychrophilum* THC02/90 strain was chosen to be deleted with the designed method because this gene is specific of this virulent strain and its product has homology with streptopain-like peptidases that are described to be virulence factors such as the streptococcal pyrogenic exotoxin (ScpB). ScpB is a critical virulence factor for invasive disease episodes caused by *S*. *pyogenes* [22–26]. Therefore, we hypothesize that the FcpB protein, which is homologous to ScpB, could have some relevance in the pathogenic process of *F. psychrophilum* THC02/90. Interestingly, although growth of the fcpB^-^ strain was not affected, a significant decrease in extracellular proteolytic activity when azocasein was used as substrate was found in comparison with the parental strain. ScpB also possesses caseinolytic activity [35]. Therefore, the reduction of this activity in fcpB^-^ strain seems to indicate that FcpB could act as an extracellular protease. However, deletion of the *fcpB* gene in *F. psychrophilum* THC02/90 had no effect on virulence since DL_50_ assays did not showed significant differences between mutant and parental strain after intramuscular injection challenges in a rainbow trout model. Similar results were obtained in the studies carried out with the *fpp1* and *fpp2* genes encoding extracellular metalloproteases since fpp1^-^ and fpp2^-^ mutants showed similar virulence levels. At this point it should be noted that intramuscular injection is the only way to get reproducible values in LD_50_ experiments with the *F. psychrophilum*, but it does not reproduce a natural infection process. Additionally, it should be considered that the genome of *F. psychrophilum* JIP02/86 encodes for, at least, 14 other extracellular proteases [9]. Therefore, this redundancy that, according to preliminary genome sequencing data, could be extrapolated to *F. psychrophilum* THC02/90 may enable some compensation, although their role on virulence should be studied in each case.

The mutagenesis method described in this work should allow relevant progress in the study of *F. psychrophilum* gene function. This strategy could be used for deleting any non-essential gene, creating mutants with multiple deletions, removing large DNA fragment containing cluster of genes, introducing point mutation in genes of interest or inserting foreign DNA fragment at the desired location in the *F. psychrophilum* genome.

## Supporting Information

S1 Information Animal experiment protocols and mortality data (Table A: Daily dead fish injected with different doses of bacterial strains). (DOCX)
