# Supplementary material for: Development of a Markerless Deletion System for the Fish-Pathogenic Bacterium Flavobacterium psychrophilum
Source: PLoS One. 2015 Feb 18;10(2):e0117969. doi: 10.1371/journal.pone.0117969 (PMC4333118; doi:10.1371/journal.pone.0117969)
Supplement: S1 Information — (DOCX) [file pone.0117969.s001.docx]

**Supporting Information**

**S1 Information. Animal experiment protocols and mortality data.**

BACKGROUND AND PURPOSE: *Flavobacterium psychrophilum* is a Gram-negative fish pathogen that causes important economic losses in aquaculture worldwide. Although the genome of this bacterium has been determined, the functional role and relative importance of genes in relation to virulence remain to be established. To investigate their respective contributions to the bacterial pathogenesis, effective tools for gene inactivation are required.

EXPERIMENTAL APPROACH: In the present study, a markerless gene deletion system has been successfully developed for the first time in this bacterium. Using this method, the *F. psychrophilum* *fcpB* gene, encoding a predicted cysteine protease homologous to *Streptococcus pyogenes* streptopain, was deleted. The developed system involved the construction of a conjugative plasmid that harbors the flanking sequences of the *fcpB* gene and an *I-SceI* meganuclease restriction site. Once this plasmid was integrated in the genome by homologous recombination, the merodiploid was resolved by the introduction of a plasmid expressing I-SceI under the control of the *fpp2* *F. psychrophilum* inducible promoter. The resulting deleted *fcpB* mutant presented a decrease in extracellular proteolytic activity compared to the parental strain. However, there were not significant differences between their LD_50_ in an intramuscularly challenged rainbow trout infection model. The mutagenesis approach developed in this work represents an improvement over the gene inactivation tools existing hitherto for this “fastidious” bacterium. Unlike transposon mutagenesis and gene disruption, gene markerless deletion has less potential for polar effects and eventually allows the mutation of virtually any non-essential gene or gene clusters.

KEY RESULTS: A new system for gene deletion in *F. psychrophilum* was developed.

CONCLUSIONS AND IMPLICATIONS: We have showed that it was possible the construction of a gene deletion system in *F. psychrophilum* using as a model the *fcpB* gene, encoding a predicted cysteine protease that was not involved in the bacterial virulence. The system could be used for deleting any non-essential gene, to create mutants with multiple deletions, to delete large DNA fragment containing cluster of genes, to introduce point mutation in genes of interest or to insert foreign DNA fragment at the desired location in the *F. psychrophilum* genome.

INTRODUCTION: *Flavobacterium psychrophilum* is the etiological agent of the bacterial cold-water disease (BCWD) and rainbow trout fry syndrome (RTFS), which particularly affects juvenile rainbow trout (*Oncorhynchus mykiss*), causing important economic losses in salmonid aquaculture worldwide. The disease mainly appears when water temperatures range between 10ºC and 14ºC and, as there is no commercial vaccine, its control requires the massive use of antibiotics.

The study of virulence factors of *F. psychrophilum* has been drastically hampered by the difficulty to genetically manipulate this organism. In view of this situation, we reasoned that the deletion of a gene specifically present in the genome of this virulent strain (*F. psychrophilum* THCO2-90) encoding for a protein homologous to the cysteine protease streptopain, a major virulence determinant in *Streptococcus pyogenes* could be the interest in order to know whether or not this gene was involved in the virulence of this bacterium. Therefore, the additional objective was then to know the implication of this gene in the pathogenic process of *F. psychrophilum* in a rainbow trout infection model.

For this purpose, we selected a rainbow trout model to carry out the experimental infections in order to calculate LD50 values. We chose this animal because it is one of the natural hosts of *F. psychrophilum.*

Therefore, one of the objectives of this study was to determine whether the *fcpB* gene is involved in the virulence of *F. psychrophilum*.

METHODS: Animal experiments were performed in accordance with the European legislation governing animal welfare. The protocol was authorized (January 12th, 2009) and supervised by the Animal Experimentation Ethics Committee of Universidad de Oviedo. All experiments were performed under tricaine methane sulfonate (MS-222) anesthesia, and all efforts were made to minimize suffering. A copy of the authorization could be send if needed.

Groups of 10 fish were challenged by intramuscular injection of 50 µl of dilutions containing 10^3^ to 10^5^ CFU of parental and fcpB mutant strains. A control group of 10 fish was injected with an equal volume of PBS. Total= 70 individuals per experiment.

Animals were randomized into the different groups (wild type challenge, mutant challenge and control). Dead fish were registered every day. The experiment was carried out in duplicate. The experimental unit was each group of ten fish.

The following table was filled out with the number of dead fish registered every day.

Table A. Daily dead fish injected with different doses of bacterial strains.

| **THC02/90**  **Exp 1** | **Dose (c.f.u.)** | **Day 1** | **Day 2** | **Day 3** | **Day 4** | **Day 5** | **Day6** | **Day 7** | **Day 8** | **Day9** | **Day 10** | **Total** |
| --- | --- | --- | --- | --- | --- | --- | --- | --- | --- | --- | --- | --- |
|  | **1,2 x 10 ^3^** | **0** | **0** | **0** | **0** | **0** | **0** | **0** | **0** | **0** | **0** | **0** |
|  | **1,1 x 10 ^4^** | **0** | **0** | **0** | **0** | **1** | **1** | **0** | **0** | **0** | **0** | **2** |
|  | **1,3 x 10 ^5^** | **0** | **0** | **4** | **1** | **5** | **0** | **0** | **0** | **0** | **0** | **10** |
| **THC02/90**  **Exp 2** |  | | | | | | | | | | | |
|  | **1,5 x 10 ^3^** | **0** | **0** | **0** | **0** | **0** | **0** | **0** | **0** | **0** | **0** | **0** |
|  | **1,6 x 10 ^4^** | **0** | **0** | **0** | **0** | **0** | **0** | **0** | **0** | **0** | **0** | **0** |
|  | **1,6 x 10 ^5^** | **0** | **0** | **3** | **2** | **5** | **0** | **0** | **0** | **0** | **0** | **9** |
| **fcpB^-^**  **Exp 1** |  | | | | | | | | | | | |
|  | **1,1 x 10 ^3^** | **0** | **0** | **0** | **0** | **0** | **0** | **0** | **0** | **0** | **0** | **0** |
|  | **1,3 x 10 ^4^** | **0** | **0** | **0** | **0** | **0** | **1** | **0** | **0** | **0** | **0** | **1** |
|  | **1, 2 x 10 ^5^** | **0** | **0** | **4** | **1** | **4** | **0** | **0** | **0** | **0** | **0** | **9** |
| **fcpB^-^**  **Exp 2** |  | | | | | | | | | | | |
|  | **1,7 x 10 ^3^** | **0** | **0** | **0** | **0** | **0** | **0** | **0** | **0** | **0** | **0** | **0** |
|  | **1,5 x 10 ^4^** | **0** | **0** | **0** | **0** | **1** | **0** | **0** | **0** | **0** | **0** | **1** |
|  | **1,5 x 10 ^5^** | **0** | **0** | **3** | **3** | **3** | **0** | **0** | **0** | **0** | **0** | **9** |
| **Control (PBS)**  **Exp 1** |  | **0** | **0** | **0** | **0** | **0** | **0** | **0** | **0** | **0** | **0** | **0** |
| **Control (PBS)**  **Exp 2** |  | **0** | **0** | **0** | **0** | **0** | **0** | **0** | **0** | **0** | **0** | **0** |

Fish were challenged by intramuscular injection of 50 µl of dilutions containing 10^3^ to 10^5^ CFU of the corresponding strain or PBS in the case of the control group. Prior to injection, fish were exposing to tricaine methane sulfonate (MS-222) as analgesic. For euthanasia an overdose of this compound was used (100 mg/l). In both cases the anesthetic was first mixed well with a small quantity of water before it was poured to the bath.

Anesthesia was applied out several minutes before each challenge experiment. Euthanasia was carried out when dying fish showed clear clinical symptoms of a terminal infection as erratic swimming or hemorrhagic ulcers. It was also applied to surviving fish at the end of the experiment (10 days post infection).

Fish were kept in 60 l tanks at 12ºC±1 in dechlorinated water. Each batch was microbiologically analyzed for potential pathogens before and during all the experimental process

Anesthesia was applied by bath immersion to minimize suffering of the animals. Overdose of this compound was used (also by bath immersion) to humanely kill both dying animals and surviving fish at the end of the experiment.

Rainbow trout (*Oncorhynchus mykiss*) fries of about 5-7 g obtained from a commercial fish farm were used in all the experiments

One hundred and ten individuals were obtained from a commercial fish farm and acclimatized for at least 48 h. Microbiological analysis indicated that the fish were free of known viral, bacterial and parasitic pathogens.

Fish were kept in 200 l tanks at 12ºC±1 in dechlorinated water until the experiment was carried out. Then, each group of ten fish was transferred to a 60 l tank with the same conditions.

Fish were maintained under starvation conditions during the experiment. The tanks were placed in a temperature (12±1ºC) controlled room. The oxygen and pH levels were also controlled.

Prior intraperitoneal injection, pain was minimized by using tricaine methane sulfonate (MS-222).

The total number of animals was 70 per experiment, ten per each experimental group.

The animals were transported in 75 l tanks with oxygen for 15 minutes periods. Statistical determination in order to obtain significant results was used. The experiment was repeated in triplicate. Animals were randomized into the different groups.

First, three groups of ten fish were challenged against the parental strain by intramuscular injection. Then other three groups were challenged against the mutant strain. Finally, the control group was injected with PBS.

LD50 was calculated according to the PROBIT method using the SPSS statistical package for Windows, establishing a 95% confidence limit.

For each test, the experimental unit was each group of ten fish.

RESULTS: The animals’ health status was checked prior the experiments by microbiological analysis. These indicated that the fish were free of known viral, bacterial and parasitic pathogens. One hundred and ten animals were used by experiment (7 groups of 10 fish). Two experiments were carried out.

Ten fish were not included in the experiment, because they were not necessary. They were also euthanized by overdose of the anesthetic agent.

The values obtained were 1.48 x 10^5^ CFU (with lower and upper 95% confidence limits with a lower bound of 9.08 x 10^4^ CFU and an upper bound of 2.51 x 10^5^ CFU), for the parental strain. Under the same conditions, fcpB- showed a LD50 value of 1.70 x 10^5^ CFU, with a lower bound of 1.01 x 10^4^ CFU and an upper bound of 2.86 x 10^5^ CFU.

DISCUSION: The aim of our study was to develop a deletion system for *F. psychrophilum* in order to generate a genetic tool for studding virulence determinants in this fish pathogenic bacterium. To carry out this procedure the fcpB gene was selected. The *fcpB* gene is predicted to encode a 394 amino-acid protein with significant homology with bacterial cysteine endopeptidases belonging to the C10 family peptidases, including the streptopain (ScpB) of *S. pyogenes*, a pyrogenic exotoxin described to be a relevant virulence factor. This feature together with the fact that the *fcpB* gene was specific of the virulent strain THC02-90 suggested that this gene could play a role in virulence. Once the gene was deleted, we considered essential to check whether its lost produced the expected attenuation in the virulence of the bacterium. LD50 experiments showed that there was no relation between the *fcpB* gene and virulence in *F. psychrophilum*.
